# A standardized protocol for microplastic and non-synthetic microfiber extraction and µFTIR identification in elasmobranch gastrointestinal tissues^☆^

**DOI:** 10.1016/j.mex.2026.103996

**Published:** 2026-06-06

**Authors:** Roberto Cruz-García, Felipe Amezcua

**Affiliations:** Instituto de Ciencias del Mar y Limnología, Unidad Académica Mazatlán, Universidad Nacional Autónoma de México. Av. Joel Montes Camarena S/N, C.P. 82040, Mazatlán, Sinaloa, México

**Keywords:** Gastrointestinal analysis, Polymer identification, Marine contamination monitoring, Density separation, Organic matter digestion, Spectroscopic characterization

## Abstract

Elasmobranch gastrointestinal tissues are a complex matrix for microplastic work: high in lipids, dense in connective tissue, and prone to losing small particles during handling. In this work, we developed a protocol that targets these problems. Tissue is digested in 20% (w/v) KOH at 40°C until the soft fraction dissolves (24–72 h); when organic residues persist, 30% (v/v) H₂O₂ is added until the solution clears to amber. The digest is then density-separated in 5 M NaCl, filtered through 1.2 µm glass fiber, sorted under a stereomicroscope, and identified by µFTIR-ATR.

Validation on eight samples spiked with 100 polyethylene reference particles (50–500 µm) gave recoveries of 90–96% (mean 93.4 ± 2.06%); the procedural blank returned 97%. From the real samples, 172 particles were recovered, mostly fibers, with cellulose as the dominant material, alongside PET, acrylic, nylon, and polypropylene. Detection was reliable down to ∼150 µm by stereomicroscopy and ∼60–70 µm by µFTIR.

The method is not designed for particles below 50 µm or polymers denser than the saline brine (∼1.2 g cm⁻³); because both require adapting the density step.

Key features of the method:

• Sequential KOH–H₂O₂ digestion handles the fat- and protein-rich matrix of shark and ray digestive tissue without altering common polymers.
• Mean recovery of 93.4% on polyethylene-spiked samples (90–96%); 97% in procedural blanks.
• µFTIR-ATR identification down to ∼60 µm, with a reliable visual sorting threshold of ∼150 µm.

## Graphical abstract

**Figure:**
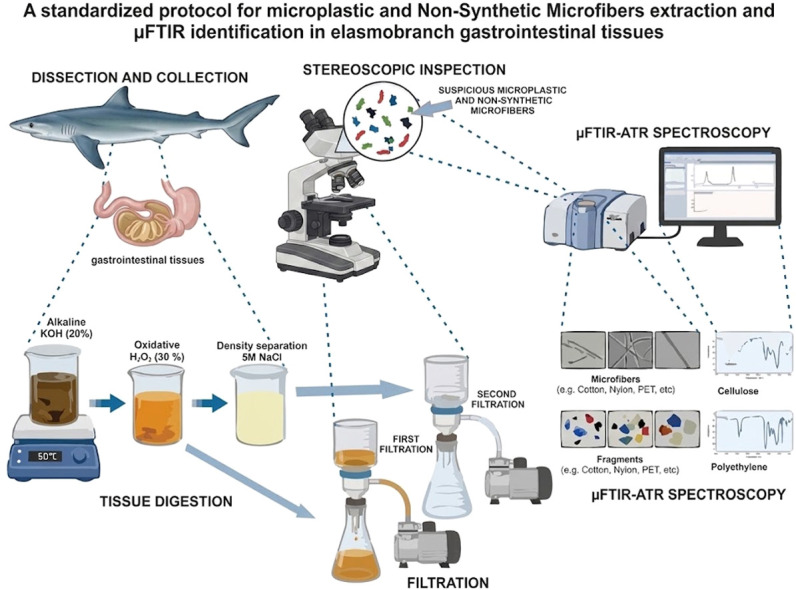

**Specifications table**

**Table:** 

**Subject area**	Environmental Science
**More specific subject area**	Microplastic extraction from biological matrices: Digestion and isolation of microplastics in elasmobranchs
**Name of your method**	A standardized protocol for microplastic and non-synthetic microfibers extraction and µFTIR identification in elasmobranch gastrointestinal tissues
**Name and reference of original method**	Adapted from existing microplastic isolation protocols using alkaline digestion and oxidative treatment (e.g., [[1], [2], [3]]) and recent methodological approaches in elasmobranchs (e.g., [4,5]), optimized for shark and ray gastrointestinal matrices.
**Resource availability**	All reagents and laboratory equipment described are commercially available. The Nicolet™ iN™10 micro-FTIR system integrates FTIR spectral libraries. Reference polyethylene particles are available from standard polymer suppliers.

## Background

Microplastics (MPs) are now reported from coastal sediments to the deep sea [6,7]. Marine animals ingest them at every trophic level, and the downstream effects, such as gut abrasion, transfer of sorbed contaminants, behavioral changes in feeding, have been documented in fish, seabirds, and mammals [8,9]. Sharks and rays sit at the top of the food web; whatever their prey carry, they inherit it [10,11]. Recent surveys have found MPs and non-synthetic microfibers (NSMs) in the digestive tracts of elasmobranchs at significant levels. But recovering these particles from those tracts is not trivial. Elasmobranch gastrointestinal tissue is fat-heavy and dense in connective material; it resists chemical digestion, and small particles that survive the lab work are easily lost during filtration. The methods currently in use, such as visual sorting, microscope picking, and KOH digestion at various concentrations, were mostly developed for teleosts, bivalves, or zooplankton, and these do not always transfer well to elasmobranchs [2,3]. Recovery rates are seldom reported; when they are, the discrepancy between studies is high, which makes cross-study comparisons difficult.

This paper addresses the issue by presenting an extraction protocol for elasmobranch gastrointestinal tissues. The protocol includes information on recovery efficiency, procedural blanks, and microscopy/µFTIR detection limits.

## Method details

The following protocol was developed to extract and identify MPs and other non-synthetic microfibers (NSMs) from elasmobranch gastrointestinal (GI) tissues efficiently. Given the complexity and high organic content of these samples, this protocol prioritizes complete digestion of the tissue, control of background contamination, and recovery of particles without chemical alteration. The workflow is divided into four main stages: sample preparation, chemical digestion, density separation and filtration, and spectroscopic identification.

### Sample preparation and handling

The gastrointestinal tract was extracted immediately after specimen collection to minimize degradation. When immediate processing was not possible, samples were stored frozen (−20°C) in pre-cleaned glass containers. The dissection was undertaken in a clean environment, under a laminar flow hood, which is the ideal procedure. All laboratory personnel wore non-shedding cotton lab coats and nitrile gloves. Synthetic clothing was avoided to reduce airborne contamination. All reagents and instruments were prepared and made ready before sample processing began.

The materials used for the extraction of MPs from elasmobranch digestive tracts were: a) Organism Dissection: Metal dissection tray, dissection kit (mosquito forceps, scalpel, dissection scissors). b) Organic Matter Digestion: 500 mL glass jars with metal lids, 60 × 15 mm glass Petri dishes, 5 mL pipette, vacuum pump, 500 mL capacity vacuum filtration apparatus, Whatman™ GF/C glass fiber filters with 1.2 µm mesh size and 47 mm diameter, Potassium Hydroxide (KOH) in a 20% solution, 30% Hydrogen Peroxide (H_2_O_2_), and 5 M (NaCl) solution. c) Filter Examination: Stereoscopic microscope fitted with a Zeiss AxioCam ERC 5s digital camera (Carl Zeiss Microscopy GmbH, Jena, Germany). d) MP Material Determination: Micro-Fourier Transform Infrared Spectroscopy using an Attenuated Total Reflectance accessory (µ-FTIR-ATR) Nicolet™ iN™ 10 equipment, including a diamond crystal (D-SlidIR) and a liquid-nitrogen-cooled mercury cadmium telluride detector.

Glassware and metal materials, including the glass fiber filters, underwent pretreatment in an oven at 400°C for 4 hours, and the dissection equipment was washed with methanol and distilled water, which had been pre-filtered using Whatman™ GF/C glass fiber filters (1.2 µm) to prevent cross-contamination.

### Dissection

The gastrointestinal tract was removed from each elasmobranch specimen by dissecting the organism. For this, a longitudinal incision was made from the cloaca to the esophagus. Both ends of the digestive tract were secured with mosquito forceps to prevent the release of the stomach contents and subsequent sample contamination. The weight of each organ was recorded. The stomach contents and tissue from the organs of interest (stomach and intestine) were placed onto metal dissection trays. Visible putative plastic particles (PPPs) were sought via visual inspection and separated from the sample for subsequent analysis. Using stainless steel instruments, the GI tract was opened, and the contents were then carefully transferred into pre-cleaned glass beakers. The internal surfaces were rinsed with filtered distilled water to recover any remaining material. The wet weight of the sample was recorded.

Practical note: Procedural blanks (e.g., filtered water exposed during sample handling) were included at every dissection to assess airborne contamination.

### Chemical digestion of organic matter

**Fig. 1:**
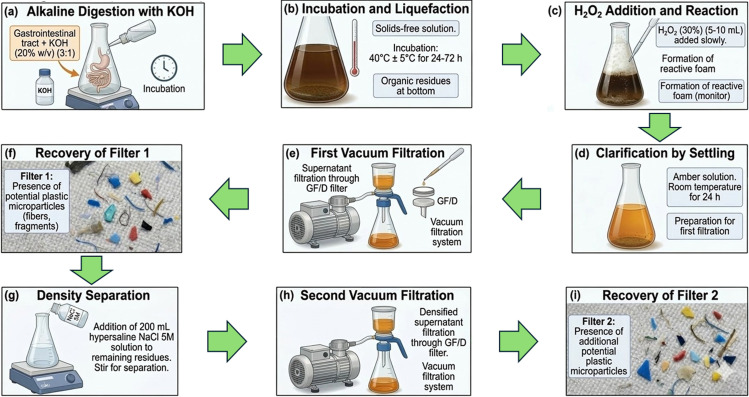
Flow chart of the laboratory work for chemical digestion, filtration, and isolation of putative plastic particles (PPPs). a) KOH is added to the digestive tract; b) sample incubation; c) H₂O₂ is added to the digestive tract; d) sample digested and ready for filtration; e) first filtration of the chemical digest; f) filter containing PPPs; g) 5 M hyper-saline solution is added and the sample is agitated; h) second filtration of the hyper-saline solution; i) filter containing PPPs.

Because of the high lipid and protein content of elasmobranch tissues, a combined digestion approach is necessary: alkaline and oxidative digestion. The method was performed on eight samples, including a blank control containing only double-distilled water. Each sample and the blank were spiked with a known number (100 particles per sample) of polyethylene (PE) reference MPs, ≈ 50-500 µm in size. The samples were placed in 500 mL glass jars. For the alkaline digestion, a solution of 20% (w/v) potassium hydroxide (KOH) was added at a ratio sufficient to fully submerge the sample. Samples were incubated at 40°C ± 5°C until no solids remained in the solution (approximately 24 to 72 h) (Fig. 1a) (Table 1). The mixture turned dark brown, indicating that the initial digestion was complete (Fig. 1b).

**Table 1:** Chemical digestion process showing the phased color change of the solution upon the addition of different reagents.

Reactive	Physical Description of the Sample
**KOH (20%) (w/v)**	a) The organic matter begins to disintegrate, taking on a dark and homogeneous color and depositing organic matter as sediment at the bottom of the glass container.
**H_2_O_2_ (30%) (v/v)**	b) The sample may react vigorously, producing abundant foam and beginning to turn a lighter color over time. It will eventually result in a transparent amber-yellow color with light-colored organic residues settling at the bottom of the container as sediment.

Following incubation and tissue dissolution, for samples with persistent organic residues, oxidative digestion was performed using hydrogen peroxide (H₂O₂, ∼30%). Here, 10 mL of 30% (v/v) H_2_O_2_ was added daily to each sample at room temperature until the solution transitioned to an amber color (approximately 24 h) (Table 1). Because high reactivity and foaming were possible, the H_2_O_2_ was administered in small, slow increments (Fig. 1c, d).

Observation: A homogeneous solution with minimal visible organic fragments indicates complete digestion. Overexposure to strong oxidants should be avoided, as it may damage certain polymers.

### Density separation

Once digestion was complete, microplastics were separated based on density. The clear fraction (amber yellow without solid residues) of the resulting solution was transferred into a separation funnel or graduated cylinder.

Approximately 200 ml of high-density saline solution (e.g., NaCl or denser alternatives if targeting heavier polymers) was added to the remaining solid fraction that settled at the bottom of the glass jar. After gently mixing, the system was left to settle (typically 12–24 hours). This difference in density causes the flotation of the PPPs present in the solid organic matter, allowing the subsequent supernatant to be filtered in the same way the clear amber fraction was (Fig. 1g, h, i). After settling, the supernatant containing low-density particles was carefully collected. The bottom sediment was left undisturbed.

Tip: Performing at least two sequential separations can improve recovery, especially for smaller particles.

### Filtration

Both collected supernatants were filtered to retain particles. Using a pipette aid, the entire supernatants were passed through a glass fiber filter (pore size 1.2 µm) employing a vacuum pump and a Whatman™ GF/C filter, which captures the PPPs (Fig. 1e, f). Filters were pre-rinsed with filtered distilled water before use. Filtration was achieved under vacuum in a closed system.

After filtration, filters were placed in covered glass Petri dishes (60 × 15 mm) and dried at room temperature.

Contamination control: Blank filters were placed in parallel, and all filters were stored covered when not in use.

### Visual sorting and preliminary classification

Dried filters were examined under a stereomicroscope. Particles were categorized based on shape (fibers, fragments, films, etc.), color, and size. Suspected MPs and NSMs were carefully isolated using metal tweezers and transferred to new glass fiber filters for further analysis.

Tip: Consistent criteria need to be used for classification to ensure reproducibility across samples and operators. Considerations to determine PPPs include small size (largest dimension ≤5mm), no cellular or organic structures visible, homogeneous color throughout the particle, and for fibers, equal thickness throughout their entire length. Particles must also show flexibility and resistance when prodded, though some may show signs of weathering [2,12].

### Polymer identification (µFTIR analysis)

Selected particles were analyzed using micro-Fourier Transform Infrared spectroscopy (µFTIR) with a Nicolet™ iN™10 micro-FTIR system. Particles were placed on appropriate substrates (e.g., ATR crystals), and the spectra were acquired in the mid-infrared range and compared against the system’s integrated FTIR reference libraries for polymer identification.

Acceptance criteria: Matches above a defined similarity threshold (commonly ≥75%) were considered reliable. Ambiguous spectra were flagged and reanalyzed.

### Quality control

Blanks: Procedural and airborne controls were included at every stage to detect any potential contamination originating from laboratory handling or airborne particles. During all stages of sample processing, including dissection, chemical digestion, filtration, and microscopic examination, a crystallization dish containing double-distilled water was placed next to the working area to act as an airborne procedural blank. These blanks were exposed to the same laboratory environment and processing conditions as the biological samples (Table 3).

To further minimize contamination, several precautionary measures were implemented. All glassware and filters were pre-combusted at 400°C; reagents and distilled water were pre-filtered through glass-fiber filters, and work surfaces and instruments were carefully cleaned prior to each processing step. Samples were kept covered during digestion and filtration to reduce the risk of airborne fiber deposition (Table 3).

Important note: Contamination is one of the primary sources of bias in microplastic studies. Strict adherence to clean lab practices is essential throughout the procedure.

### Data recording and reporting

The following information was recorded for each sample:

• Number and type of particles.
• Polymer composition.
• Size range.
• Recovery rates and blank corrections.

Standardized reporting facilitates comparison across studies and strengthens the reliability of ecological interpretations.

### Final remarks

The protocol was developed and validated for elasmobranch gastrointestinal tissue. It can be transferred to other organisms with comparable lipid content and connective-tissue density, although the digestion time and the density of the flotation solution will probably need to be adjusted. For tissues with markedly different compositions, such as soft-bodied invertebrates or vertebrates with leaner digestive tracts, laboratory personnel should re-optimize reagent concentrations and incubation times before they apply the method quantitatively. The protocol is not intended as a universal procedure, and any extension beyond elasmobranchs should be supported by an independent recovery test.

## Method validation

Validation addressed four parameters: recovery efficiency, reproducibility across replicates, polymer integrity after chemical treatment, and the detection limit of each analytical step.

Eight tissue samples and one procedural blank (double-distilled water only) were processed under the full protocol. Each was spiked with 100 polyethylene reference microplastics in the 50–500 µm size range before digestion, so that recovery could be measured against a known input. Recovered PE was counted by stereomicroscopy and confirmed by µFTIR-ATR. Recovery was calculated as:

$$Recovery\;\left(\%\right)=\frac{Number\;of\;MPs\;Recovered}{Number\;of\;MPs\;Introduced}\times \;100$$

Across the eight tissue samples, recovery ranged from 90% to 96%, with a mean of 93.4 ± 2.06% (Table 2). The procedural blank returned 97%. Variation between replicates remained below 6%, which indicates that the digestion and filtration steps performed consistently under the conditions tested.

**Table 2:** Method validation metrics for the microplastic extraction protocol applied to elasmobranch gastrointestinal tissues.

Validation parameter	Procedure	Replicates (n)	Result	Interpretation
Recovery efficiency	Samples spiked with 100 polyethylene (PE) reference particles (50–500 µm) prior to digestion	8	95%, 90%, 96%, 92%, 93%, 91%, 94%, 96%.	Mean recovery = 93.4±2.06%
Blank recovery	Procedural blank spiked with 100 PE particles	1	97%	Confirms minimal particle loss during digestion and filtration
Reproducibility	Independent digestion replicates processed under identical conditions	9	Variation <6% among replicates	Indicates consistent digestion and filtration performance
Digestion efficiency	Visual assessment of tissue dissolution after KOH and H₂O₂ digestion	9	Complete dissolution within 24–72 h	Confirms suitability for dense GI tissues
Detection limit (microscopy)	Visual inspection using stereomicroscope (10–40 ×)	–	≥150 µm	Reliable detection threshold for particle sorting
Polymer identification	µFTIR-ATR spectral matching	–	Match threshold ≥75%	Reliable polymer confirmation

**Table 3:** Quality assurance and contamination control procedures implemented during the microplastic extraction protocol.

Step of protocol	Potential contamination source	Control measure implemented	Outcome
Sample dissection	Airborne fibers from clothing or laboratory environment	Use of pre-cleaned metal instruments and glass trays; dissection area cleaned with filtered water	No particles detected in procedural blanks
Reagent preparation	Contamination from water or reagents	All liquids pre-filtered through GF/C filters (1.2 µm)	Prevented introduction of external particles
Glassware and filters	Residual particles in laboratory materials	Glassware and filters combusted at 400°C for 4 h	Eliminated background contamination
Sample handling	Airborne deposition during digestion and filtration	Samples kept covered; laboratory surfaces cleaned before processing	No contamination detected
Airborne contamination monitoring	Fibers settling during processing	Open crystallization dish containing filtered water used as airborne blank	No MPs detected in blanks
Filtration system	Cross-contamination between samples	Filtration apparatus rinsed with ethanol and filtered distilled water between samples	No carry-over detected

Polymer integrity was assessed by re-examining the recovered PE particles under µFTIR-ATR. The characteristic absorbance bands of polyethylene were preserved in every spectrum, and no visible deformation or surface alteration was observed under the stereomicroscope. Chemical degradation during processing was therefore considered negligible for polyethylene; the same conclusion cannot be extrapolated to more sensitive polymers without separate testing.

Besides the spiked PE, 172 anthropogenic particles were recovered from the eight tissue samples: 139 fibers and 33 fragments. Fibers were the dominant morphology. Black was the most frequent color, followed by gray. µFTIR identification returned cellulose as the most common material, with PET, acrylic, nylon, and polypropylene also present.

Detection limits varied by analytical step. Stereomicroscopic sorting was reliable down to approximately 150 µm; below that size, particles became difficult to separate from organic debris with confidence. µFTIR-ATR lowered the limit to about 60–70 µm, but spectral matching became unreliable for smaller particles, where the signal-to-noise ratio drops and library matches lose specificity. Particles below ∼60 µm therefore fall outside the working range of the protocol and would require complementary techniques such as Raman micro-spectroscopy.

Soft-tissue digestion was complete in all eight replicates within 24 to 72 hours. The variation in digestion time was because of differences in the lipid and connective-tissue content among samples rather than to inconsistency in the digestion step itself.

## Limitations

This protocol has several limitations that users should consider. The NaCl brine (∼1.2 g cm⁻³) does not float polymers denser than seawater, such as PVC or PET, which tend to settle with the sediment fraction; the additional filtration of that fraction recovers most of them but not all, and labs working specifically on dense polymers should switch to a denser separation medium (e.g., NaI or ZnCl₂). The sequential KOH–H₂O₂ digestion was tested on polyethylene and did not alter its FTIR signature, but the conclusion does not extend to more labile polymers, especially after long oxidative exposure; for those, the H₂O₂ step should be shortened or omitted. Visual sorting was reliable from ∼150 µm upward, and µFTIR-ATR from ∼60 µm; particles smaller than that cannot be quantified with this protocol and require techniques such as Raman microspectroscopy. Finally, even with strict contamination control, non-synthetic microfibers can be introduced from the laboratory air during handling, which sets a practical floor on how low the blank counts can go.

## Ethics statements

The gastrointestinal tracts used for the development and validation of this protocol were obtained from dead specimens sourced from local fish markets. Therefore, no live animal handling or specific ethical approval from an Institutional Animal Care and Use Committee (IACUC) was required for this methodological study.

## Declaration of generative AI and AI-assisted technologies in the manuscript preparation process

During the preparation of this work, the authors used ProWritingAid to check grammar and improve the readability of the English text. After using this tool, the authors reviewed and edited the content as needed and take full responsibility for the content of the publication.

## CRediT authorship contribution statement

**Roberto Cruz-García:** Validation, Formal analysis, Data curation, Writing – original draft. **Felipe Amezcua:** Supervision, Project administration, Conceptualization, Funding acquisition, Writing – original draft.

## Declaration of competing interest

The authors declare that they have no known competing financial interests or personal relationships that could have appeared to influence the work reported in this paper.

## Data Availability

Data will be made available on request.
